# Pulmonary thromboembolism due to immune-mediated hemolytic anemia in a cat: A serial study of hematology and echocardiographic findings

**DOI:** 10.3389/fvets.2022.930210

**Published:** 2022-08-11

**Authors:** Tomohiko Yoshida, Ahmed S. Mandour, Manami Sato, Miki Hirose, Rina Kikuchi, Norihiro Komiyama, Hanan A. Hendawy, Lina Hamabe, Ryou Tanaka, Katsuhiro Matsuura, Akiko Uemura

**Affiliations:** ^1^VCA JAPAN-Mitaka Veterinary Group, Tokyo, Japan; ^2^Department of Veterinary Surgery, Tokyo University of Agriculture and Technology, Tokyo, Japan; ^3^Department of Animal Medicine (Internal Medicine), Faculty of Veterinary Medicine, Suez Canal University, Ismailia, Egypt; ^4^Department of Clinical Veterinary Medicine, Obihiro University of Agriculture and Veterinary Medicine, Hokkaido, Japan

**Keywords:** cat, pulmonary thromboembolism, immune-mediated hemolytic anemia, echocardiography, hematobiochemical

## Abstract

Pulmonary thromboembolism (PTE) secondary to immune-mediated hemolytic anemia (IMHA) is rarely diagnosed in cats. In this report, a 3-year-old cat was referred to our private hospital with dyspnea, anorexia, and anemia. On the thoracic radiography, cardiac enlargement and pulmonary edema were noted. Echocardiography revealed right ventricular and right atrium enlargement with mild tricuspid regurgitation (tricuspid regurgitation velocity 3.28 m/s). A thrombus was recognized in the main pulmonary artery on the right parasternal short-axis view. Blood examination suggested regenerative anemia and autoagglutination. The findings suggested immune-mediated hemolytic anemia and PTE. Antithrombotic therapy (dalteparin) and immunosuppressive therapy (prednisolone) were administered under oxygen supplementation in the ICU cage. After treatment, regenerative anemia and right-heart failure were improved. During follow-up, the cat remained hemodynamically stable, and the owner reported no cardiac-related clinical signs. Further blood examination confirmed the anemia was improved. Prednisolone was discontinued on Day 56, and the cat continues in good health, administered only mycophenolate mofetil. The clinical outcome of PTE secondary to the IMHA throughout 100 days of periodical observation was reported.

## Introduction

Immune-mediated hemolytic anemia (IMHA) in cats is considered to occur less frequently than in dogs (1). Early reports suggested that IMHA is more likely to be secondary to underlying disease in cats. However, more recent studies suggest that the proportion of cats with primary IMHA is higher, similar to that reported in dogs (2–7). Clinically, as in dogs, IMHA causes rapid erythrocyte destruction by autoimmunity, resulting in mucosal pallor, jaundice, hemoglobinuria, and abdominal distention due to splenomegaly, which can be fatal if progressing rapidly (7). In canine IMHA, disseminated intravascular coagulation syndrome (DIC) and pulmonary thromboembolism (PTE) are the most common causes of death, and antithrombotic therapy has been stated to play a significant role in prognosis (8, 9). In cats, two cases of PTE secondary to IMHA were reported (10). However, the occurrence of thrombosis in the pulmonary artery and the necessity of antithrombotic therapy in the treatment protocol have not been clarified. In the present study, we diagnosed IMHA and secondary PTE in a 3-year-old cat with respiratory distress and visible mucosal pallor. The cat was treated for IMHA with prednisolone and mycophenolate mofetil, and with antithrombotic therapy. The case description, steps of diagnosis, the treatment strategy, and patient follow-ups for 100 days are reported here.

## Case presentation

A 3-year-old spayed munchkin (Body weight 2.6 kg) was referred with the primary complaints of respiratory distress, loss of energy, loss of appetite, and visible mucosal pallor. She had already been treated with antibiotics at another hospital, and blood tests revealed anemia and auto-agglutination of the red blood cells. Haemoglobinuria was noted on urination, stool observations were unremarkable, with no evidence of melena or fresh blood. Body temperature on admission was 37.4°C, with a heart rate of 180 beats/min, and a respiratory rate of 60 breaths/min. The patient presented jaundiced on visible mucosa, light pigmentation of the nasal speculum, no enlarged peripheral lymph nodes, and a capillary refill time of <2 s. The cat showed a palpable femoral artery and a systolic apical murmur on the right chest wall. The patient had a subcutaneous hemorrhage at the site of blood sampling at another hospital. A complete blood count (CBC), blood coagulation tests, and hematology and biochemistry test was performed as part of a systemic screening examination (Table 1, day 0). Blood typing was performed in consideration of blood transfusion. To exclude the infectious causes of hemolytic anemia, testing for common hemolytic anemia inducing pathogens was also performed. Because of profound dyspnea, the thorax and abdomen were evaluated by X-rays and ultrasonography.

**Table 1:** Hematological and biochemical analysis in a cat with immune-mediated hemolytic anemia.

**Variables**	**Examination intervals**							**Reference range (11, 12)**
	**Unit**	**Day 0**	**Day 2**	**Day 14**	**Day 28**	**Day 35**	**Day 100**	
WBC	10^3^/μℓ	21.2	9.8	20.0	6.6	24.5	17.6	7.73–18.6
RBC	10^6^/μℓ	1.58	5.20	6.4	8.00	7.36	9.11	5.9–11.2
HGB	g/dℓ	3.8	8.2	8.5	11.2	10.4	12.5	8.2–15.3
HCT	%	12	28.2	31.1	33.9	33.2	35.1	24–46
MCV	fL	79.7	50.4	42.6	42.4	44.0	46.1	35.9–53.1
MCHC	g/dl	31.7	30.4	32.9	33.0	33.2	34	28.1–35.8
Reticulocyte	10^3^/μℓ	78.2	132.0	10.3	13.0	5.12	4.4	3.0–50.0
Reticulocyte	%	5.0	2.5	0.2	0.2	0.7	0.5	0.2–1.0
PLT	10^3^/μℓ	43	19.2	16.3	22.6	26.3	20.7	13.0–62.6
PT	second	9	8	-	-	-	-	6.0–8.6
APTT	second	31	32	-	-	-	-	12.0–51.3
Fib	mg/dl	108	202	-	-	-	-	120–400
D-dimer	μg/ml	1.22	-	-	-	-	-	<2.0
FeLV		Negative	-	-	-	-	-	-
FIV		Negative	-	-	-	-	-	-
Coombs test		Negative	-	-	-	-	-	-
ANA		Negative	-	-	-	-	-	-
FeVBPs		Negative	-	-	-	-	-	-
TP	g/dℓ	6.5	6.7	6.8	7.0	7.5	7.0	5.4–7.8
Alb	g/dℓ	2.7	2.8	3.2	3.6	3.6	3.4	2.1–3.3
GLU	mg/dℓ	99	180	148	132	131	161	64–152
GGT	IU/l	4	-	-	2	3	-	1–7
BUN	mg/dℓ	43.7	34.6	20.3	17.2	21	20.6	15–37
Cre	mg/dℓ	1.26	0.95	0.71	0.43	0.52	0.51	0.8–1.8
ALT	IU/ℓ	50	-	62	>2000	121	77	19–90
AST	IU/ℓ	66	-	66	>1000	61	57	12–45
ALP	IU/ℓ	75	-	34	155	110	90	9–50
Na	mEq/ℓ	155	149	152	154	153	155	145–159
K	mEq/ℓ	3.7	4.1	4.0	4.6	4.2	4.0	3.0–4.8
Cl	mg/dℓ	114	104	107	104	105	105	111–125
IP	mg/dℓ	6.0	3.6	3.0	4.3	4.2	3.7	2.2–6.5
Ca	mg/dℓ	9.6	9.0	10.5	12.5	11.5	11.1	8.0–11.1
T-Bil	mg/dℓ	1.0	0.1	0.1	1.1	0.1	0.1	0.1–0.5

WBC, white blood cells; RBC, red blood cells, HGB, hemoglobin; HCT, hematocrit; PLT, platelet count; PT, prothrombin time; APTT, activated prothrombin time; Fib, fibrinogen; FeLV, feline lentivirus; FIV, feline immune virus; TP, total protein; Alb, albumin; GLU, glucose; GGT, gamma-glutamyl transferase; BUN, blood urea nitrogen; Cre, creatinine; ALT, alanine aminotransferase; AST; aspartate transferase; ALP, alkaline phosphatase; Na, sodium; K; potassium; Cl, chloride; IP, inorganic phosphorus; Ca, calcium; T-Bil, total bilirubin; ANA, antinuclear antibody; FeVBPs, feline vector born pathogens.

**Figure 1:**
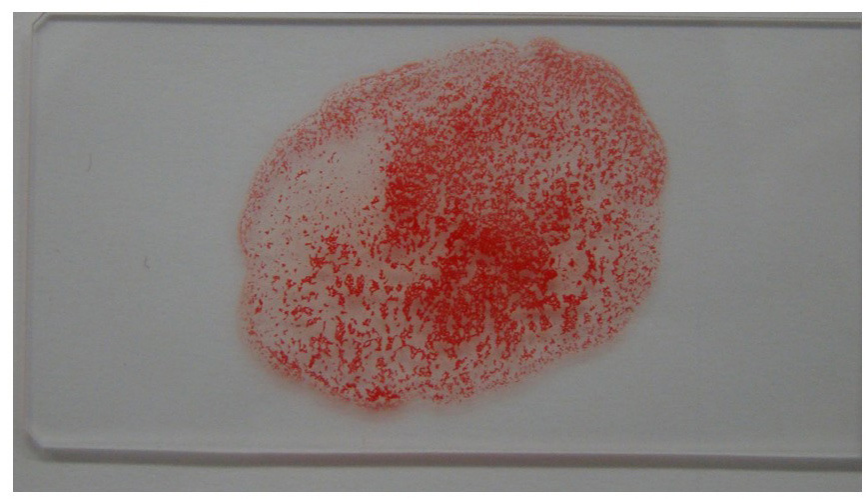
Results of in-saline slide agglutination test (SAT). Self-agglutination was positive.

**Figure 2:**
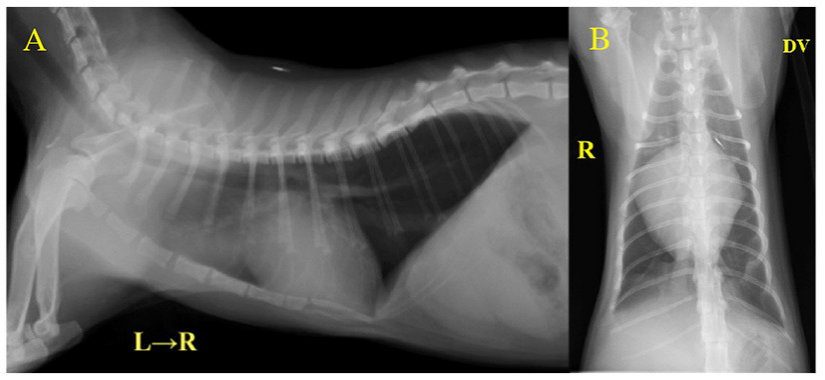
Thoracic radiography at initial examination. **(A)** Right lateral view, **(B)** Dorsoventral view. The increased pulmonary opacity was observed in the bilateral anterior lobe of the lungs. VHS = 9.4 v and CTR = 81% indicated a clear cardiac enlargement.

**Figure 3:**
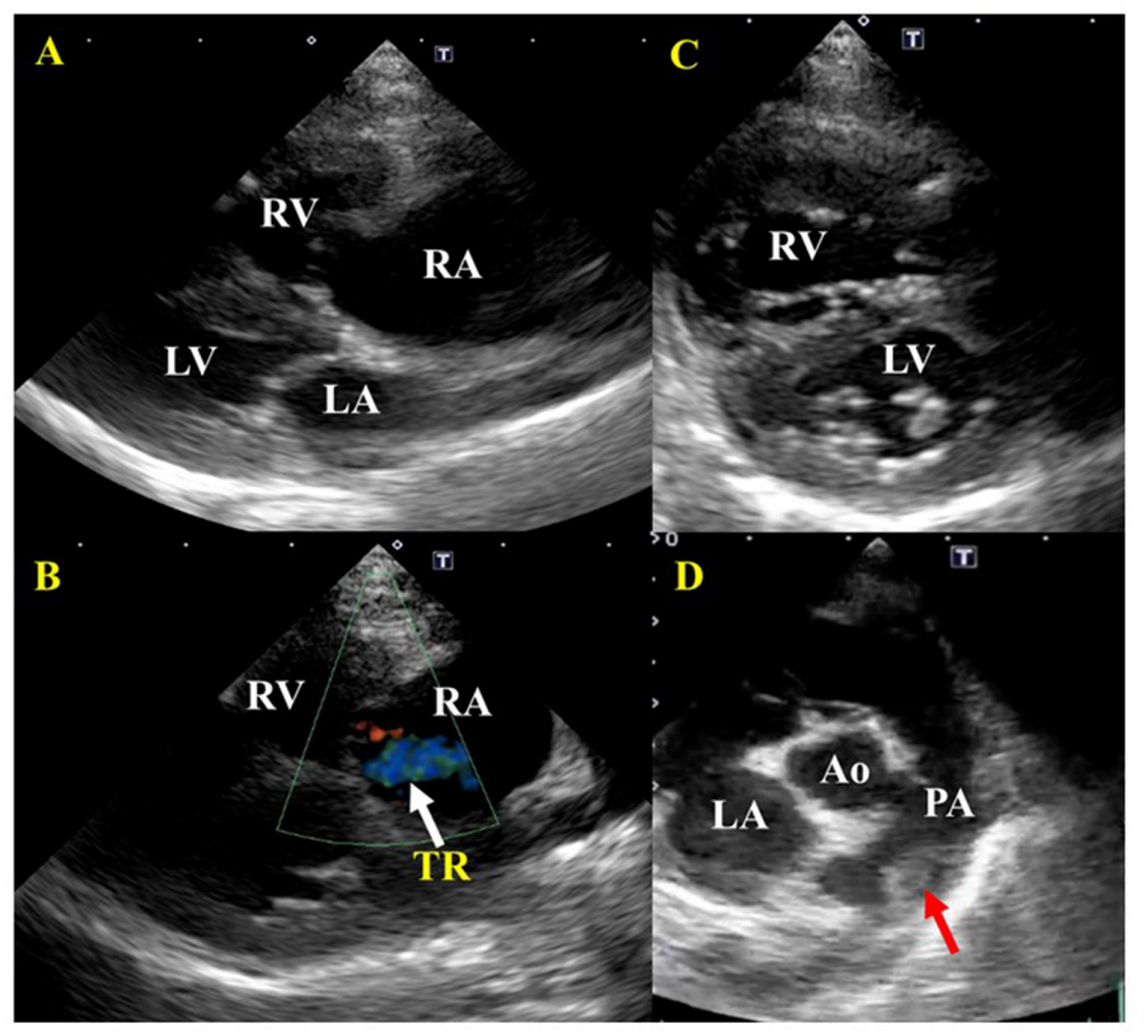
Echocardiographic images before treatment of a cat diagnosed with IMHA and pulmonary thromboembolism. **(A)** Right parasternal long-axis view. Enlargement of the right atrium and right ventricle were found. There was no dysplasia of the tricuspid valve, patent ductus arteriosus, atrial septal defect, or ventricular septal defect. **(B)** Right parasternal long-axis view (Color Doppler imaging). Tricuspid regurgitation was recognized. **(C)** Right parasternal short-axis view (Apical level). The flattening of the interventricular septum was observed. **(D)** Right parasternal short-axis view (Basal level). A thrombus was found between the main pulmonary artery and the bifurcation of the right and left pulmonary arteries. RA, right atrium; RV, right ventricle; LA, left atrium; LV, left ventricle; PA, pulmonary artery. Red arrows indicate a thrombus; white arrows indicate tricuspid regurgitation (TR).

Laboratory examination revealed severe regenerative anemia, increased white blood cell count, and decreased platelet count (ProCyte Dx Hematology Analyzer, IDEXX Laboratories, Japan). In addition, the auto-agglutination of red blood cells on the slide was observed (Figure 1). Blood chemistry tests showed elevated total bilirubin and phosphorus concentration (DRI-CHEM 7000V, Fujifilm, Japan). Blood coagulation tests are also shown in Table 1 (PT, APTT, Fib). The blood fibrinolytic system test revealed a normal D-dimer level (Fujifilm Monolith, reference value: 0–2.0 μg/ml). The blood type was determined to be type B. There was no evidence of infectious disease-causing hemolytic anemia: negative feline vector-borne pathogens (FeVBPs), feline leukemia virus antigen (FeLV), and feline immunodeficiency virus antibody (FIV) tests (IDEXX Laboratories, Japan). Also, the antiglobulin antibody (Coombs test, Fujifilm Monolith, Japan) and the anti-nuclear antibody test (IDEXX Laboratories, Japan) were negative. On thoracic and abdominal radiographs, the right lateral view showed the increased pulmonary opacity in the anterior lung lobes and the disappearance of the ventral waist of the cardiac shadow, suggesting right heart enlargement and pulmonary edema (Figure 2A). In the dorsoventral view, the interlobar fissure in the posterior lung lobe was clear, suggesting pleural effusion (Figure 2B). Abdominal radiography showed no significant alteration. Echocardiography revealed an enlarged right ventricle and right atrium with tricuspid regurgitation (TR flow 3.28 m/s) in the right parasternal long-axis view (Figures 3A,B). The right parasternal short-axis view showed flattening of the interventricular septum on B-mode images (Figure 3C). In the right parasternal short-axis view at the base of the heart, dilatation of the main pulmonary artery was observed (PA/Ao 1.1), and the pulmonary arterial flow velocity was 0.8 m/s with a shortened acceleration time to ejection time ratio (AT/ET 0.25). Hyperechoic structure, which was suspected thrombus, was observed in the pulmonary artery bifurcation (Figure 3D). Early diastolic mitral inflow (E wave) was 0.5 m/s. Right ventricular fractional area change (RV-FAC) was 18%, obtained by tracing the RV endocardial border at end-diastole and end-systole from the left parasternal long axis 4-chamber view. The right atrial area (RAA) was 2.75 cm^2^, measured by tracing from the lateral aspect of the tricuspid annulus to the septal aspect, excluding the area between the leaflets and annulus, following the RA endocardium at the end of the ventricular systole. A small amount of pleural effusion was observed, however, thoracentesis was not performed due to the low platelet count and bleeding tendency. There was no evidence of short-circuit disease or structural abnormality of the tricuspid valve. Abdominal ultrasonography showed a trace amount of ascites which was difficult to collect. There were no other significant findings, and no tumor lesions were observed.

In the present report, blood tests and imaging studies revealed no underlying disease, infection, or disease-causing regenerative anemia. The diagnosis of primary IMHA was made based on the presence of auto-agglutination as a sign of immune mediated destruction as well as the presence of jaundice and haemoglobinuria. These two signs suggesting the prescence of hemolysis. Our diagnosis also was confirmed as IMHA by the absence of another underlying cause for the anemia. Besides, observation of a structure in the pulmonary artery was thought to be a thrombus, potentially a complication of PTE. As the patient presented with severe anemia and respiratory distress, a CT scan was not performed. Instead, a blood transfusion and improvement of respiratory symptoms were prioritized.

**Figure 4:**
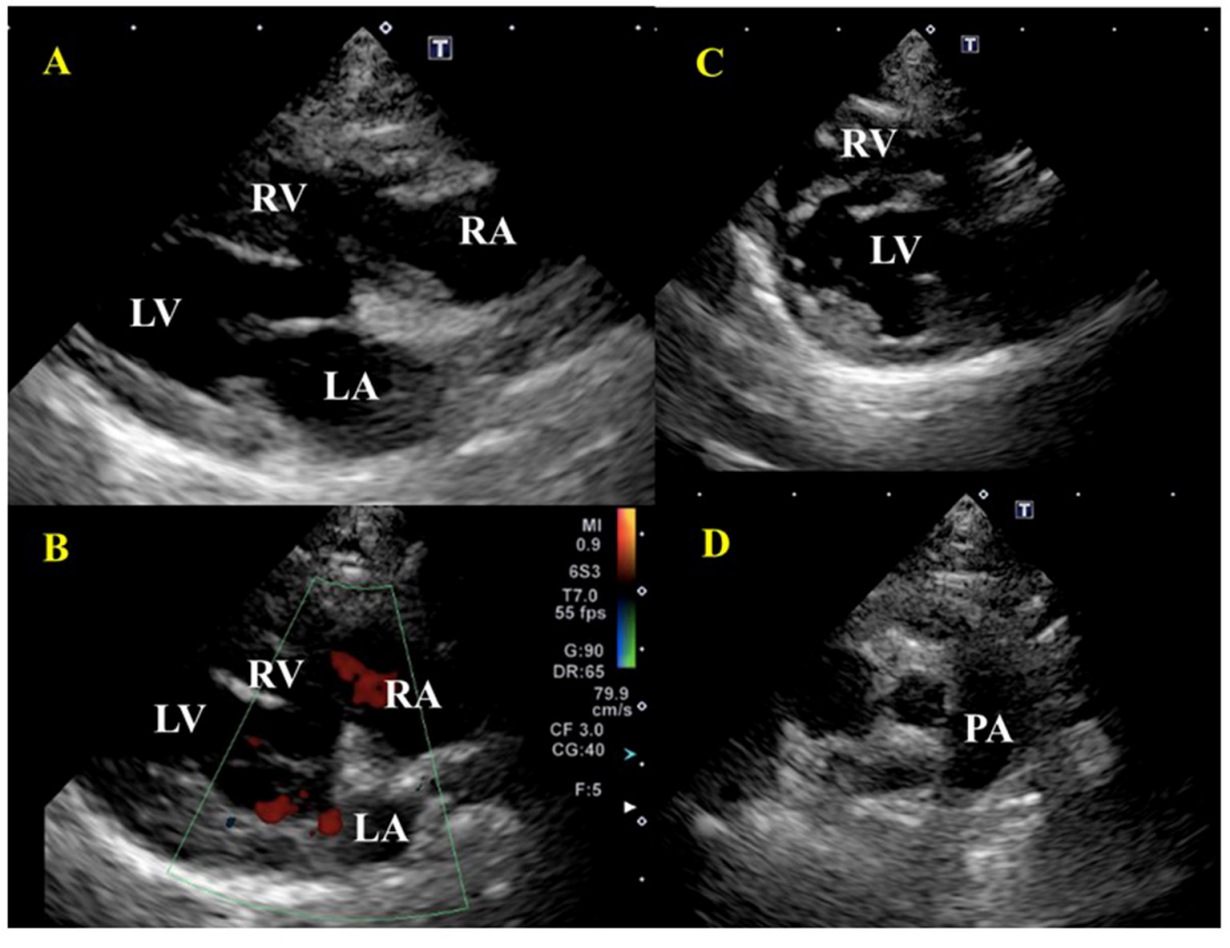
Echocardiographic images after treatment of a cat diagnosed with IMHA and pulmonary thromboembolism on Day 21. **(A)** Right parasternal long-axis view. There was no enlargement of the right atrium and right ventricle. **(B)** Right parasternal long-axis view (Color Doppler imaging), tricuspid valve regurgitation had disappeared. **(C)** Right parasternal short-axis view (Apical level). Right ventricular enlargement and flattening of the ventricular septum disappeared. **(D)** Right parasternal short-axis view (Basal level). A thrombus in the pulmonary artery had disappeared. RA, right atrium; RV, right ventricle; LA, left atrium; LV, left ventricle; PA, pulmonary artery.

The treatment protocol was initiated based on the previously published report including blood transfusion, diuretic, anticoagulant, immunosuppressant, antibiotic, and antiacid (4). The blood transfusion consisted of 40 ml of whole fresh blood, given to the recipient over 24 h to avoid rapid volume loading. Since the right ventricular volume increased after the transfusion, risking a worsening respiratory condition, furosemide 0.5 mg/kg IV (Lasix Injection, Nichi-Iko Co., Ltd., Japan) was administered at the beginning of the transfusion; the respiratory condition stabilized after a few minutes. Echocardiography and blood pressure were measured every 6 h in the first 48 h. Dalteparin sodium, a low molecular weight anticoagulant, 100 IU/kg TID SC (IV Fragmin, Kissei Pharmaceutical Company, Nagano, Japan) was administered for PTE; prednisolone 4 mg/kg/day SC (prednisolone injection solution, Kyoritsu Pharmaceutical Company, Tokyo, Japan) for IMHA treatment, and famotidine (antiacid) 1 mg/kg BID IV (Gaster, LTL Corporation, Tokyo, Japan) was administered to protect the digestive tract. Enrofloxacin 5 mg/kg SID SC (Baytril, Bayer Yakuhin, Ltd., Japan) was also administered to treat possible causes of feline infectious anemia. After the transfusion, on day two, the anemia improved. The respiratory condition was enhanced a little but respiratory frequency remained high, necessitating the administration of furosemide 0.5 mg/kg SC BID (Gaster, LTL Corporation, Tokyo, Japan). On day four, the respiratory condition was improved and the patient was able to feed independently with increased activity. During hospitalization, she continued to receive dalteparin sodium, prednisolone, famotidine, and enrofloxacin. On Day 14, as the respiratory symptoms had calmed down and anemia had not progressed, the patient was treated with prednisolone 4 mg/kg/day PO (prednisone 5 mg tablet, Shionogi, Japan); furosemide 0.5 mg/kg BID PO (Furosemide tablet 10 mg, Nipro K.K., Japan); lansoprazole 1 mg/kg BID PO (Lansoprazole, Daiko Pharmaceutical, Japan); rivaroxaban 1 mg/kg SID PO (Igzarelto tablets 10 mg, Bayer Yakuhin, Japan); leflunomide 2 mg/kg SID PO (Arava tablets 10 Sanofi K.K., Japan), and the patient was discharged from hospital. On Day 21, echocardiography showed a reversible reduction in the right ventricle and right atrium size, disappearance of the TR (Figures 4A,B), no flattening of the interventricular septum (Figure 4C), and improvement of RV-FAC (39.1%) and RAA (0.74 cm^2^). Besides, the thrombus in the pulmonary artery had disappeared and the patient's respiratory condition became stable (Figure 4D). At this moment, the dose of furosemide was reduced to 0.5 mg/kg SID. On Day 28, the visible mucosa showed a slightly jaundiced color. Blood chemistry tests showed elevated ALT, AST, ALP, GGT, and T-Bil. The cat's activity was decreased but no decrease in appetite was observed. There was no increase in serum amyloid A (<3.75) (DRI-CHEM IMMUNO AU10V, Fujifilm, Japan) or progression of anemia. Since leflunomide or the high-dose prednisolone was considered a cause of the liver disorder, leflunomide was withdrawn and mofetil mycophenolate 10 mg/kg BID (CellCept suspension spray 31.8%, Chugai, Japan) was additionally prescribed. The prednisolone was reduced to 3 mg/kg/day, and acetic acid ringer's solution (1 ml/kg/h) (Fuso Pharmaceutical Industries, Osaka, Japan) was administered under hospitalization. On Day 35, an improvement in bilirubin and liver enzymes was observed. On Day 44, further improvement of bilirubin and liver enzymes was observed. On Day 56, anemia was completely resolved, and the dose of prednisolone was reduced to 2 mg/kg/day. Further, the dose of prednisolone was decreased to 0.5 mg/kg every 2–4 weeks, and on Day 100, both prednisolone and reversaloxaban were withdrawn. At present, the cat is continuing her IMHA treatment solely with mycophenolate mofetil 10 mg/kg BID.

## Discussion

The IMHA diagnostic criteria in the ACVIM consensus statement include the saline agglutination test (SAT) and the direct antiglobulin test (Coombs test). According to the ACVIM guidelines, biomarkers of immune destruction including flow cytometry are evaluated, and at least two of these must be present to make a definite diagnosis. The presence of one of the above and at least two haemolytic findings (when only one indication of immune mediated destruction is present) such as hyperbilirubinemia/hyperbilirubinuria, hemoglobinemia, hemoglobinuria, or erythrocyte ghosting with no abnormalities in the hepatobiliary system, can also support the diagnosis of IMHA (1). In this case, the diagnosis of IMHA was made presumptively based on the presence of jaundice, regenerative anemia, and hemoglobinuria, as well as the auto-agglutination of red blood cells. Since various diseases cause hemolytic anemia, it may be important to fulfill the diagnostic criteria, especially in cats, where the incidence of primary IMHA has historically been considered to be lower than in dogs. In dogs, thromboembolism has been reported to occur in association with the onset of IMHA, either by the activation of coagulation factors or by the formation of erythrocyte auto-aggregates (13, 14). There are, however, few reports on IMHA and thromboembolism in cats, and the incidence of thromboembolism is still unknown. In the present case, PTE secondary to IMHA was suspected in the patient presented with respiratory distress; echocardiography showed a thrombus in the right and left pulmonary artery branches, and right heart enlargement disappeared after several days of treatment with antithrombotic drugs. The sensitivity and specificity of D-dimer in the diagnosis of thrombosis in cats are lower than those in dogs (15, 16), and in this case, D-dimer was normal despite the presence of a thrombus in imaging studies. Therefore, a comprehensive diagnosis of thrombosis in cats, including imaging and blood tests, is necessary. If thrombosis is largely suspected from the results of imaging tests, it is worthwhile to start antithrombotic therapy if there is no bleeding tendency or findings that suggest blood loss. As treatment for PTE in this patient, low molecular weight heparin was administered subcutaneously on admission, as the patient was unable to take oral medication due to her worsening respiratory condition, and rivaroxaban was prescribed at the time of discharge from the hospital because of improved general condition. In addition, a diuretic was administered to reduce the right ventricular capacitance load, and the patient's respiratory condition improved to a good standard (17). It has been reported that a good course of treatment for PTE in dogs has been achieved by administering antithrombotic drugs and oxygen inhalation (16). As such, we decided to follow the same protocol by administering antithrombotic drugs under oxygen inhalation. In this case, factor Xa inhibitor, reversaloxaban, was the antithrombotic therapy of choice. Although there are fewer reports on the use of rivaroxaban for thrombosis in cats than clopidogrel and other drugs, we chose rivaroxaban as the patient was relatively small in stature; the smaller tablet size of rivaroxaban made it easier for the owner to administer the drug. However, the dosage of rivaroxaban for cats has not been clearly defined, and setting the dosage could be a significant challenge in the future (18). The PTE is a cause of pulmonary edema by inducing pulmonary hypertension (19). This may have been the case in our patient since her respiratory urgency was reduced in response to increased impermeability in the lung fields and diuretics on imaging examination. In such patients in poor general condition, diuretics may decrease blood pressure and lead to a potentially fatal situation; as such, it is recommended that diuretics be administered while measuring blood pressure and monitoring the right and left ventricular cavity size, and the left ventricular inflow tract waveform (E waveform) by echocardiography, to maintain hemodynamic status. The treatment of pulmonary edema could be effectively performed while preserving the patient's health. Pulmonary vasodilators were also considered in the current case but were not administered as they are not a fundamental treatment for addressing hypertension. For the treatment of IMHA in cats, we used prednisolone and leflunomide as immunosuppressive therapy. On Day 28 of treatment, leflunomide was withdrawn after a possible leflunomide-related adverse reaction was observed, i.e., elevated liver enzymes and bilirubin. The patient quickly improved, although ALP remained persistently elevated. Since there are few reports on adverse reactions to immunosuppressive drugs in cats (20), it is unclear whether leflunomide causes such adverse reactions specifically in cats or just in this case, but it should be used with caution. In addition, the possibility of hepatic injury due to prednisolone cannot be ruled out, and regular monitoring of liver enzyme levels is necessary when high-dose prednisolone is used. Since the cause of low creatinine, and low chloride also were unclear, these values also should be monitored. Although no serious adverse reactions have occurred with immunosuppressive therapy using mycophenolate mofetil, the lack of large-scale studies in cats suggests the need for further careful monitoring of these patients.

In the future, when long-term remission is achieved, mofetil mycophenolate will also be withdrawn.

## Conclusion

We reported a case of primary IMHA complicated by PTE in a cat. Blood transfusion, immunosuppressive therapy, antithrombotic therapy, and diuretics are effective treatment strategies for patients with IMHA and PTE, but detailed hemodynamic monitoring, including echocardiography and blood pressure measurement, is necessary during the induction, due to significant hemodynamic changes.

## Data availability statement

The raw data supporting the conclusions of this article will be made available by the authors, without undue reservation.

## Ethics statement

Ethical review and approval was not required for the animal study, given there was only one case, for which written informed consent for participation was obtained from the owners. Written informed consent was obtained from the owners for the participation of their animals in this study.

## Author contributions

TY, AM, and MS designed the report and drafted the manuscript. KM, MH, and RK acquired and analyzed the data. NK, LH, and AU critically reviewed the manuscript. HH and RT summarized in this report. All authors contributed to the article and approved the submitted version.

## Conflict of interest

The authors declare that the research was conducted in the absence of any commercial or financial relationships that could be construed as a potential conflict of interest.

The handling editor HS, declared past co-authorships with the author, AM.

## Publisher's note

All claims expressed in this article are solely those of the authors and do not necessarily represent those of their affiliated organizations, or those of the publisher, the editors and the reviewers. Any product that may be evaluated in this article, or claim that may be made by its manufacturer, is not guaranteed or endorsed by the publisher.
